# Dietary Fiber Intake Regulates Intestinal Microflora and Inhibits Ovalbumin-Induced Allergic Airway Inflammation in a Mouse Model

**DOI:** 10.1371/journal.pone.0147778

**Published:** 2016-02-12

**Authors:** Zhiyu Zhang, Lei Shi, Wenhui Pang, Wenwen Liu, Jianfeng Li, Haibo Wang, Guanggang Shi

**Affiliations:** 1 Department of Otolaryngology-Head and Neck Surgery, Shandong Provincial Hospital Affiliated to Shandong University, Jinan, P.R. China; 2 Shandong Institute of Otolaryngology, Shandong Provincial Key Laboratory of Otology, Jinan, P.R. China; 3 Department of Otolaryngology-Head and Neck Surgery, Affiliated Eye, Ear, Nose, and Throat Hospital, Fudan University, Shanghai, China; Universidade Federal do Rio de Janeiro, BRAZIL

## Abstract

**Background:**

Recently, academic studies suggest that global growth of airway allergic disease has a close association with dietary changes including reduced consumption of fiber. Therefore, appropriate dietary fiber supplementation might be potential to prevent airway allergic disease (AAD).

**Objective:**

We investigated whether dietary fiber intake suppressed the induction of AAD and tried to elucidate the possible underlying mechanisms.

**Methods:**

The control mice and AAD model mice fed with 4% standard-fiber chow, while low-fiber group of mice fed with a 1.75% low-fiber chow. The two fiber-intervened groups including mice, apart from a standard-fiber diet, were also intragastric (i.g.) administrated daily with poorly fermentable cellulose or readily fermentable pectin (0.4% of daily body weight), respectively. All animals except normal mice were sensitized and challenged with ovalbumin (OVA) to induce airway allergic inflammation. Hallmarks of AAD were examined by histological analysis and ELISA. The variation in intestinal bacterial composition was assessed by qualitative analysis of 16S ribosomal DNA (rDNA) content in fecal samples using real-time PCR.

**Results:**

Low-fiber diet aggravated inflammatory response in ovalbumin-induced allergic mice, whereas dietary fiber intake significantly suppressed the allergic responses, attenuated allergic symptoms of nasal rubbing and sneezing, decreased the pathology of eosinophil infiltration and goblet cell metaplasia in the nasal mucosa and lung, inhibited serum OVA-specific IgE levels, and lowered the levels of Th2 cytokines in NALF and BALF, but, increased Th1 (IFN-γ) cytokines. Additionally, dietary fiber intake also increased the proportion of Bacteroidetes and Actinobacteria, and decreased Firmicutes and Proteobacteria. Levels of probiotic bacteria, such as *Lactobacillus* and *Bifidobacterium*, were upgraded significantly.

**Conclusion:**

Long-term deficiency of dietary fiber intake increases the susceptibility to AAD, whereas proper fiber supplementation promotes effectively the balance of Th1/Th2 immunity and then attenuates allergic inflammatory responses significantly, as well as optimizes the structure of intestinal microbiota, which suggests potential for novel preventive and therapeutic intervention.

## Introduction

Allergic airway diseases (AAD), such as allergic rhinitis, asthma, and so forth, are reversible and chronic atopic disorders, leading to substantial global financial and medical burden [1,2]. Recent evidence suggests that the upper and lower airways share common pathological mechanisms, characterized by Th2-like inflammatory response, such as eosinophil infiltration and goblet cell metaplasia in subepithelial mucosa, as well as increased serum levels of allergen-specific immunoglobulin (Ig) E [3–5].

Epidemiologic surveys suggest that the incidence of allergic rhinitis and asthma increased worldwide, especially in the West [1,6]. Interestingly, studies have increasingly found that this increase of allergic diseases was associated closely with dietary changes, especially chronic high-fat and low-fiber diets over the past decades [7,8]. According to ‘microflora hypothesis’ and relevant studies [9–13], long-term deficiency of fiber intake caused great variation in microbial community composition and altered the normal immunity, which contributed to the risk of developing allergic airway diseases or other inflammatory diseases. As yet, the precise mechanisms underlying the relationship still remain unclear.

Dietary fiber is a complex carbohydrate consisting of soluble fiber (pectin) and insoluble fiber (cellulose). As an essential nutrient, it has proved a crucial role in maintaining human health, with the prevention and protection actions for various human diseases [14–17]such as colon cancer, diabetes, cardiovascular and other diseases. Recently, Trompette’s study [18]suggests that fermentable fiber changes the composition of the gut microbiota, consequently by the fermentation of certain species of bacteria, leading to physiologically active byproducts (SCFAs). As the main of fiber metabolite, SCFAs shape the immunological environment and influence the severity of allergic inflammation in asthmatic mouse, which are thought to elicit their effects through binding to endogenous receptors GPR41.The latest findings from Thorburn [19]showed that high-fiber or acetate-feeding led to marked suppression of allergic asthma, by enhancing T-regulatory cell numbers and function, as well as imparts on their adult offspring an inability to develop AAD. Although with embedded study, the immunomodulatory effects of dietary fiber on AAD are known gradually, current understandings of this correlation are limited to allergic asthmatic- inflammation in lung.

To our knowledge, no study has been conducted to elucidate the role of dietary fiber in allergic inflammation in the upper airway. Therefore, herein we first establish a new mouse model with allergic rhinitis and asthma, then used three types of dietary fiber intervention to modulate allergic inflammation, and assessed its efficacy and the underlying mechanisms. We provide experimental evidence supporting the beneficial effect of dietary fiber against AAD, whereby to preventing the development of allergic respiratory inflammation.

## Materials and Methods

### Animals and Reagents

Specific pathogen-free (SPF) three-week-old female Balb/c mice (11-13g) were obtained from Animal Centre of Shandong University, P.R. China and housed under standard SPF conditions with sterile food and autoclaved water supplied by Animal Experiment Center of the Provincial Hospital Affiliated with Shandong University. Animal protocols were approved by the Animal Care Committee of Shandong University (NO. ECAESDUSM20123011).

Mice were randomly assigned to feed either a standard-fiber chow (4% content) or a low-fiber chow (1.75% content) that we purchased from KEAOXIELI FEED. Co., LTD (Beijing, China).When studying the effect of dietary fiber content, in addition to standard-fiber diet, animals were given an extra fiber supplementation of soluble pectin (Sigma-Aldric, p9135, USA) or insoluble cellulose (Sigma-Aldric, c6288, USA). Ovalbumin (Sigma-Aldrich, A5503, USA) and aluminum hydroxide (Thermo Scientific Imject Alum, 77161, USA) were used to induce Th2-type allergic responses in respiratory airway.

Wright-Giemsa and Periodic Acid Stiff-AlcianBlue (PAS-AB) as staining reagents were purchased from Beijing Solarbio Science & Technology Co., Ltd (Beijing, China). Mice OVA-specific serum IgE levels were determined by OVA-specific IgE Antibody Assay Kit (Chondrex, 3010, USA). The levels of IL-4, IFN-γ and IL-10 in BALF and NALF were tested with Mouse Enzyme Linked Immunosorbent Assay (ELISA) Kits (eBioscience, 88–7711, USA). Total bacterial DNA was extracted from fecal samples by using QIAamp DNA Stool Mini Kit (qiagen, 51504, Germany). The specific16SrDNA-targetedprimers mentioned for quantitative reverse transcription polymerase chain reaction (qRT-PCR), were designed and synthesized by Sangon Biotech Co., Ltd (Shanghai, China).

### Study Design for Dietary intervention, Sensitization and Challenge

**Fig 1:**
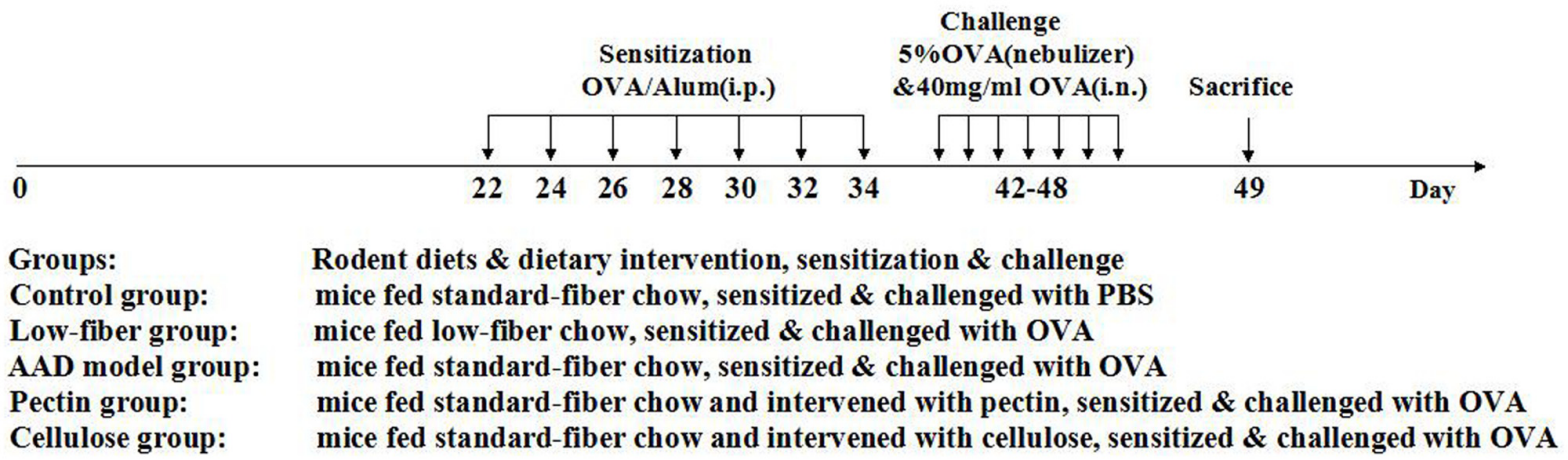
Experimental protocol. Mice were fed with different rodent diets, and then sensitized and challenged with OVA or PBS, as described previously in Materials and methods. Mice were divided into five different groups. The fiber intake and gastric volume all were within the safety and tolerance range of mice. There were no significant differences between normal group of mice and experimental group of mice by the evaluation of health condition, including body weight, fur and animal activity during the treatment.

Balb/c mice were randomly allocated into five groups (Fig 1): (A) Control group of mice fed with standard-fiber chow, sensitized and challenged with PBS instead. (B) Low-fiber group of mice fed with low-fiber chow, sensitized & challenged with OVA. (C) AAD model group of mice fed with standard-fiber chow, sensitized & challenged with OVA. (D) Pectin supplement group of mice fed with standard-fiber chow and supplemented daily with soluble pectin via gastric gavage, sensitized & challenged with OVA. (E) Cellulose supplement group of mice fed with standard chow and supplemented with insoluble cellulose, sensitized & challenged with OVA. All experimental and control groups consisted of 10 animals.

The detail gavage protocol was as following: without anesthesia, mice were intragastrically (i.g.) administrated with 400~800μl aqueous suspension of fiber powder three times every day, with the final concentration attaining the level of 40mg/ml. The daily consumption of fiber was set to 0.4% of daily body weight of mice. During the phase of allergen induction, mice were sensitized and challenged with ovalbumin to induce allergic inflammation in respiratory airway. On days 22, 24, 26, 28, 30, 32 and 34, all animals apart from the normal group were sensitized by intraperitoneal (i.p.) injection with 40 mg OVA and 2 mg aluminum hydroxide in 200 ml volume PBS. Hereafter on days from 42 to 48, mice were challenged with 5% aerosolized OVA for 30 min by inhalation, and then instilled intranasally (i.n.) with 20μl OVA (40mg/ml) each day. Mice in the normal group were sensitized and challenged with PBS only. After 24 h of the final challenge, all animals were sacrificed using a lethal dose of 10% chloral hydrate and then the tissue samples were collected and detected, as described in previous studies.

### Measurement of Allergic Symptoms

The frequency of nose rubbing and sneezing behaves per mice was counted immediately for 10 minutes after last ovalbumin atomization in a blinded manner as previously described [20].

### Cell Counts for Nasal Lavage Fluid (NALF) and Bronchoalveolar Lavage Fluid (BALF)

After 24 h of the final challenge, all mice were sacrificed and the nasal sections and the lungs were perfused with 1.2 ml PBS containing 1% fetal bovine serum (FBS) for the collection of NALF and BALF. Lavage fluids were centrifuged at 2500 rpm for 7 min at 4°C and lavage supernatant was separated and stored for further analysis. Lavage cells were resuspended in 150μl PBS and then counted with a Hemocytometer. For classification and counting, smear preparations were made and stained by Wright-Giemsa. The inflammatory cells were differentiated into monocytes, eosinophils, lymphocytes, and neutrophils according to standard morphology [21], and counted at least 300 cells at × 400 magnification with a light microscope (Leica, USA).

### Hematoxylin and Eosin (HE) Staining for Nasal Mucosa and Lung

After 24 h of the final allergen challenge, mice were sacrificed and the nasal tissues and lung were removed and then fixed in paraformaldeyde solution (4%inPBS) for 24 h. After fixation, nasal tissues still need to be decalcified with 10% EDTA for seven days. All tissue samples were embedded and prepared into paraffin sections with the thickness of 4–5μm. After staining with hematoxylin-eosin (HE), Eosinophils in nasal subepithelial mucosa were counted by using a light microscope at ×400 magnification and inflammation scores in lung tissue were assessed by a reproducible scoring system as previously described. Based on the levels of peribronchial and perivascular eosinophilic inflammation across main bronchus, scores were ranged from 0 to 3; a value of 0 was adjudged for no inflammation, a value 1 for occasional cuffing with inflammatory cells, a value 2 for thin layer of inflammatory cells surrounding most bronchi or vessels, and a value 3 for a thick layer of inflammatory cells surrounding most bronchi or vessels. At least 5 tissue sections of per mouse were selected and assessed in a randomly ordered, blinded fashion [22].

### Alcian Blue and Periodic Acid Schiff (AB-PAS) Staining of the Nasal Mucosa and Lung

In order to accurately evaluate the severity of goblet cell metaplasiain nasal mucous and bronchial epithelium, tissue sections were observed with AB-PAS staining. The goblet cells as AB-PAS positive cells were counted with a microscope and the percentages assayed from at least five tissue sections of per mouse in a blinded fashion [23].

### ELISA of Serum OVA-specific IgE

After 24 h of the final OVA challenge, a blood sample was withdrawn by heart puncture to extract serum for further determination of OVA-specific IgE levels with ELISA kit. The linear range of this assay for detection of anti-OVA IgE levels was 0.4–25 ng/ml.

### ELISA of IL-4, IFN-y, IL-10 and IL-2 in Lavage Fluid

The expression of cytokines IL-4, 1L-10 and IFN-γ in both NALF and BALF were assessed by ELISA testing, following kit’s brochures. The standard curve range for Mouse IL-4, IL-10 and IFN-γ were 4–500, 30–4000 and 15–2000 respectively.

### Bacterial DNA Isolation from Mouse Feces

Mice Feces were harvested with 2 ml perfectly clean eppendorf tube under sterile conditions 2 h before sacrifice and stored immediately in liquid nitrogen until processing for DNA isolation. Then total microbacteria DNA was isolated from feces using the Qiamp DNA Mini Kit according to the manufacturer’s instructions, which was either used directly for quantitative PCR.

### Quantitative PCR of Intestinal Bacterial DNA

To ascertain the composition of the bacterial phyla present in feces of the mice that were fed with different diets, isolated bacterial DNA was submitted to quantitative PCR and amplified using previously described primers.

(Pan-bacteria: forward: 5′-GCAGGCCTAACACATGCAAGTC-3′ and reverse: 5′-CTGCTGCCTCCCGTAGGAGT-3′;Bacteroidetes: forward:5′-CRAACAGGATTAGATACCCT-3′ and reverse: 5′-GGTAAGGTTCCTCGCGTAT-3′; Firmicutes: forward: 5′-TGAAACTYAAAGGAATTGACG-3′ and reverse: 5′-ACCATGCACCACCTGTC-3′; Actinobacteria: forward: 5′-TACGGCCGCAAGGCTA-3′ and reverse: 5′-TCRTCCCCACCTTCCTCCG-3′; Gammaproteobacteria: forward: 5′-TCGTCAGCTCGTGTYGTGA-3′ and reverse: 5′-CGTAAGGGCCATGATG-3′;*Bifidobacterium Genus*: forward: 5’-GCGTGCTTAACACATGCAAGTC-3’ and reverse: 5’-CACCCGTTTCCAGGAGCTATT-3’;*Lactobacillus* group: forward: 5’-AGCAGTAGGGAATCTTCCA-3’ and reverse: 5’-CACCGCTACACATGGAG-3’;*Escherichia coli subgroup*: forward: 5′-CATGCCGCGTGTATGAAGAA-3′ and reverse: 5′-CGGGTAACGTCAATGAGCAAA-3′;*Enterococcus spp*. forward: 5′-CCCTTATTGTTAGTTGCCATCATT-3′ and reverse: 5′-ACTCGTTGTACTTCCCATTGT-3′;).

Then 25μl PCR reactions were set up containing 2 μg of template DNA, 12.5 μl SYBR Green reaction mix (TaKaRa), 0.5 μl of each primer at a concentration of 10 μM and 9.5μl of nuclease-free water. Quantitative PCR was performed on the CFX96 Touch Real-Time PCR Detection System using the following conditions: one cycle at 95°C for 3 min, then 40 cycles at 95°C for 15 s and 61.5°C for 30 s and 70°C for 20 s, followed by a dissociation stage at 65°C for 31 s and cycles of 5 s starting at 65°C, raising 0.5°C per cycle, to obtain melting curves for specificity analysis.

### Statistical Analysis

The statistical analyses were performed with SPSS v.19.0. Graph generation and statistical analyses were performed using Prism version 5.0. Student-Newman-Keuls test or a Mann-Whitney U test was applied for analysis of experimental data, and *P*< 0.05 was considered as significant.

## Results

### Dietary Fiber Content Suppresses the Frequency of Allergic Symptoms

**Fig 2:**
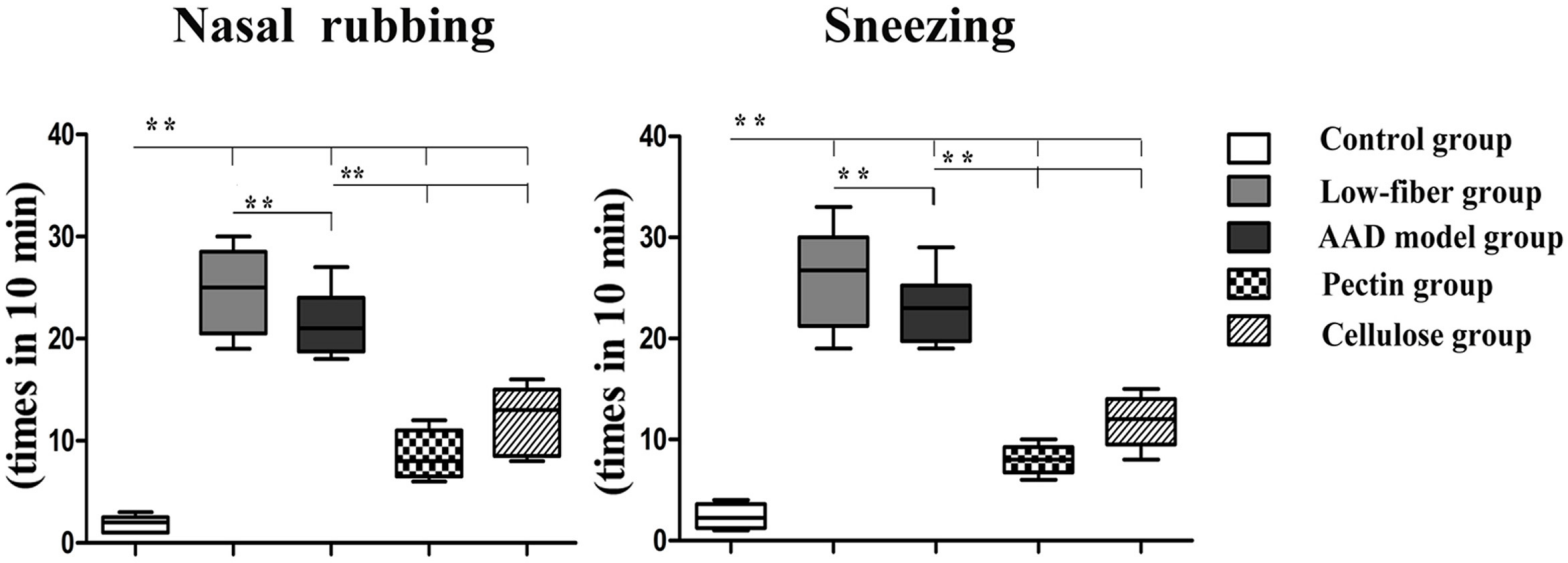
Altered allergic response. Compared with the AAD model group, the control group showed no or fewer allergic symptoms, whereas the low-fiber group showed remarkable frequency of nasal rubbing and sneezing. The frequency was significantly decreased with dietary fiber intervention. Data are shown by box and whisker plots, with whisker ends indicating minimal and maximal values and horizontal bars representing medians, n = 10; *p<0.05, **p<0.01 as conducted.

In order to determine the role of dietary fiber in allergy, we first measured the frequency of allergic symptoms per mouse[24]. The incidence of nasal rubbing and sneezing in individual mice in the AAD model group was increased significantly (both *p*<0.01), compared with that in the control group, along with more severe symptoms in low-fiber group (both *p*<0.01) (Fig 2). Interestingly, we also found that the pectin and cellulose content significantly inhibited the frequency of allergic symptoms (*p*<0.01).

### Dietary Fiber Content Decreases OVA-induced Inflammation in NALF and BALF

**Fig 3:**
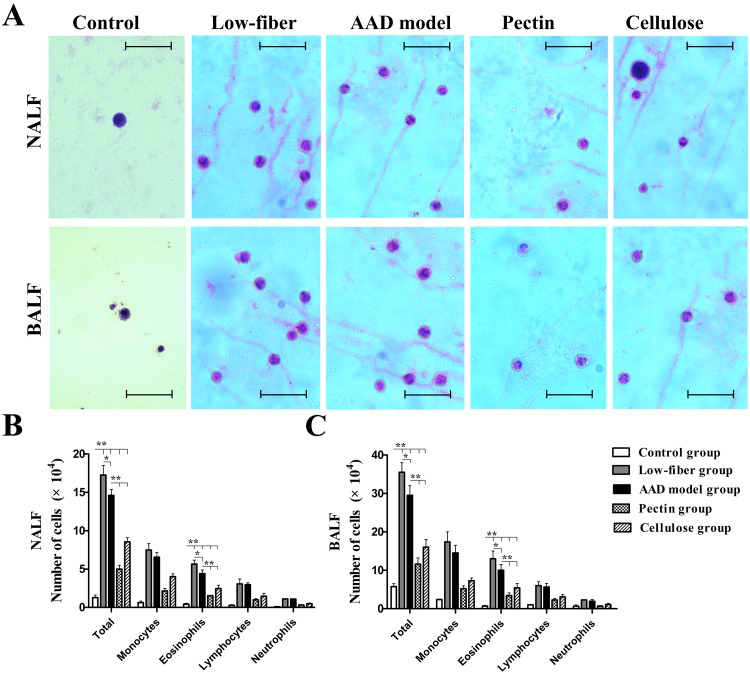
Absolute numbers of inflammatory cells in 1.2 ml of NALF and BALF at 24 h after final challenge. Total inflammatory cells and eosinophils in NALF (A) and BALF (B) were significantly inhibited by dietary fiber, whereas low-fiber diet aggravated the inflammatory response of AAD. Scale bars, 50μm.Each bar represents the mean cell number ± standard error of the mean (SEM), n = 10; *p<0.05, **p<0.01 as conducted.

We counted the inflammatory cells obtained from NALF and BALF 24 h after the final challenge (Fig 3A–3C). Increased total inflammatory cells as well as eosinophils in NALF and BALF were detected in the AAD model group than in the control group (all *p*<0.01), along with increased numbers of other related cell types (monocytes, lymphocytes and neutrophils). However, the results showed that long-term low-fiber diet increased the numbers of inflammatory cells(*p*<0.05), compared with AAD models fed with standard-fiber chow, while dietary fiber supplement reduced total cells and eosinophil cell numbers markedly both in NALF and BALF (both *p*<0.01) (Fig 3). Interestingly, the decrease in inflammation was more robust in mice supplemented with extra fermentable fiber content (pectin) than non-fermentable fiber content (cellulose) (*p*<0.01) (Fig 3). All the data suggested that dietary fiber content potently suppressed allergic symptoms.

### Dietary Fiber ameliorates OVA-induced Upper and Lower Allergic Airway Inflammation

**Fig 4:**
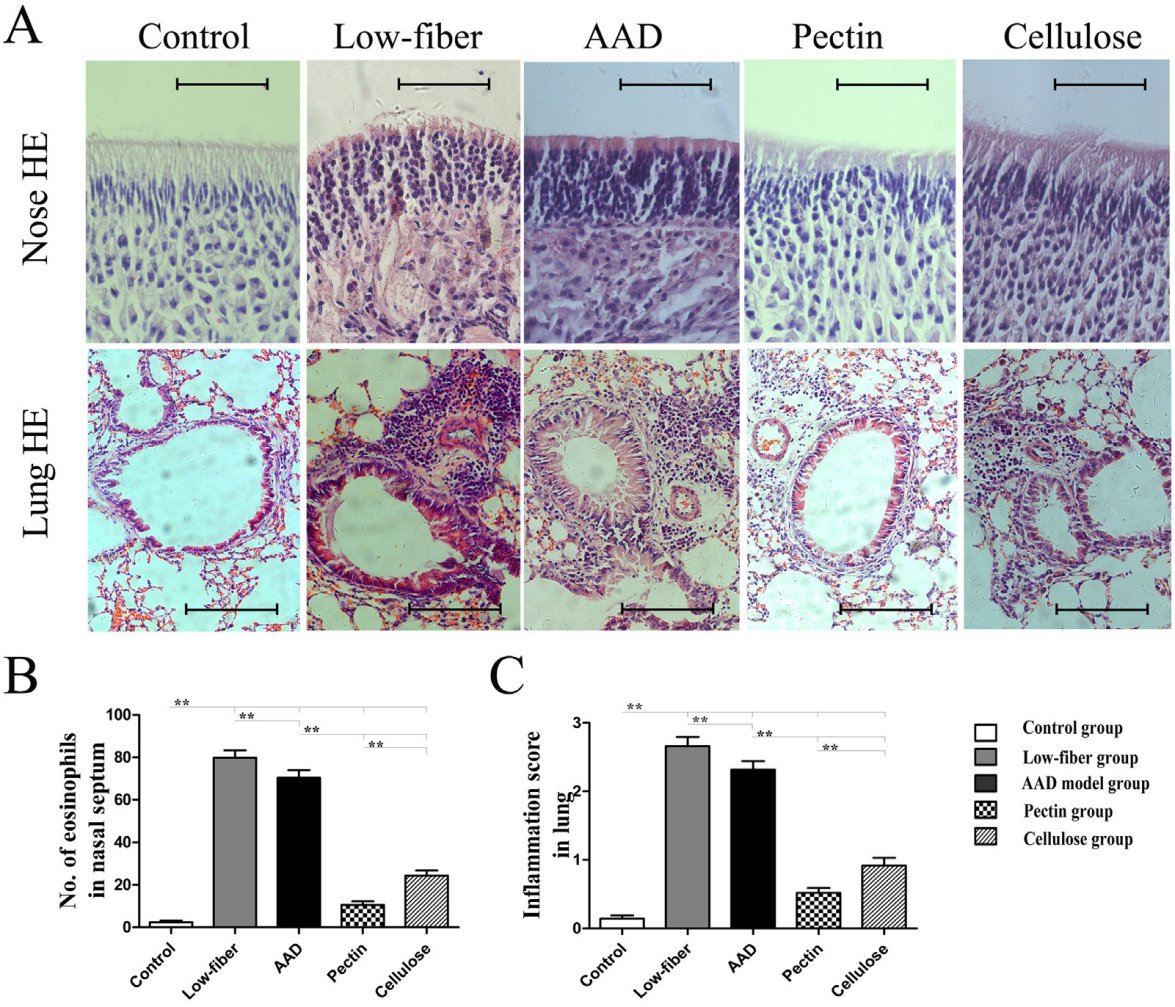
Eosinophil inflammation assessed with hematoxylin and eosin (HE)-stained tissue sections of the nasal mucosa and lung. Original magnification was × 400 for nose and × 200 for lung (A). Numbers of eosinophils in the nasal mucosa (B) and inflammation scores of the lung (C) were counted to verify the altered inflammation among groups. Eosinophil infiltration was significantly higher in low-fiber group than in the AAD model group. Interestingly, dietary fiber intervention drastically suppressed eosinophil inflammation. In addition, numbers of eosinophils in pectin group were lower than in the cellulose group. Scale bars, 200 μm for Nose; scale bars, 500 μm for Lung. Data expressed as mean ± SEM, n = 10; *p<0.05, **p<0.01 as conducted.

**Fig 5:**
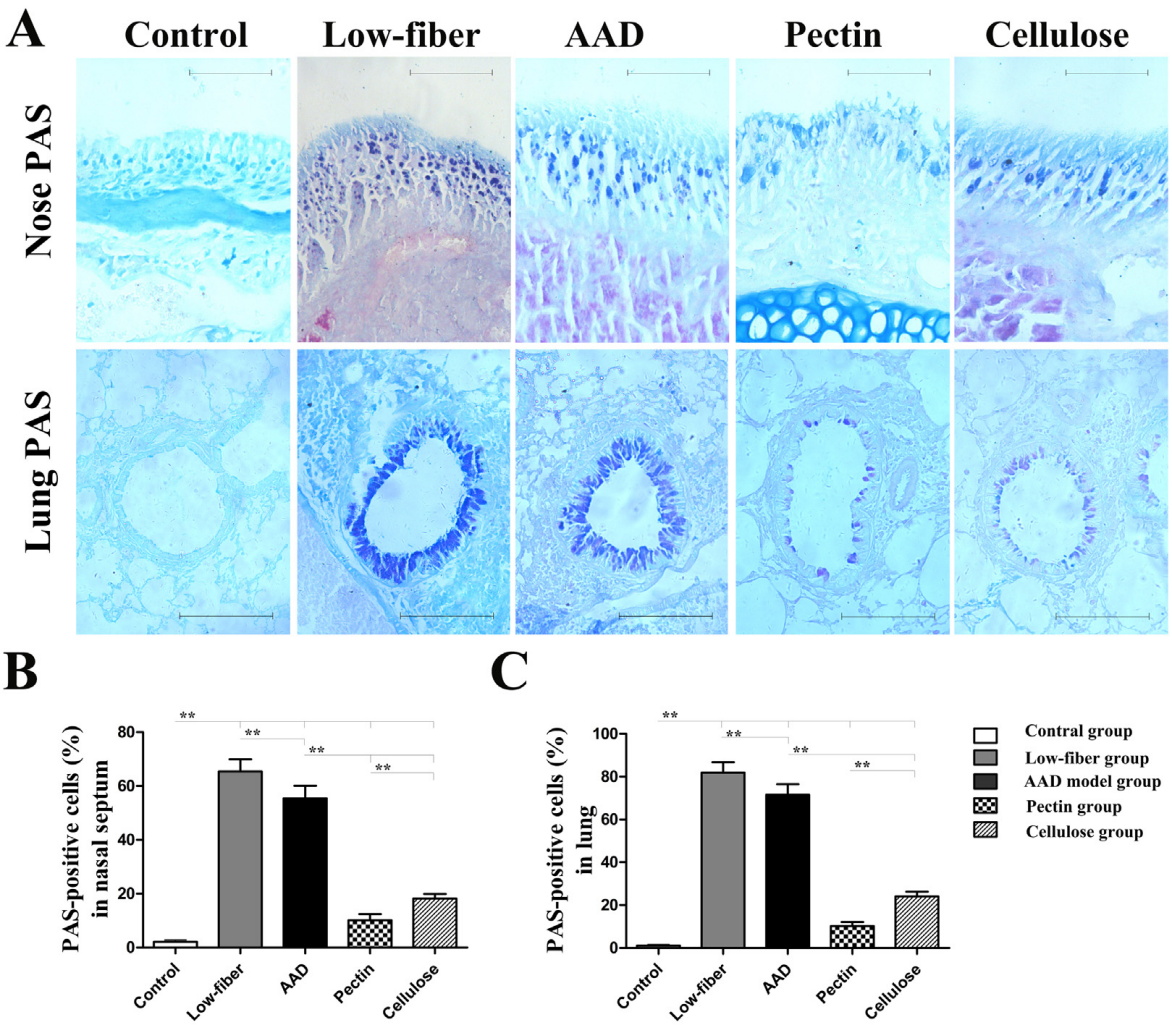
Goblet cell metaplasia inalcian blue-periodic acid Stiff (AB-PAS)-stained tissue sections of the nasal mucosa and lung. Original magnification was × 400 for nose and × 200 for lung (A). Goblet cells were counted as the positively stained blue cells. Percentages of goblet cell metaplasia were calculated from the total cell numbers counted around the nasal mucosa (B) and the lung (C). Goblet cell metaplasia was relatively minor in mice fed with dietary fiber. Scale bars, 200 μm for Nose; scale bars, 50 μm for Lung. Data are expressed as mean ± SEM, n = 10;*p<0.05, **p<0.01.

Furthermore, we examined the altered allergic airway inflammation using histological analysis of eosinophil inflammation and goblet cell metaplasia in the nasal mucosa and lung. Compared with the standard-fiber diet, insufficiency of dietary fiber intake aggravated allergic inflammation (Fig 4). However, in fiber-intervened groups there was a significant suppression of eosinophil infiltration (all *p*<0.01) (Fig 4) and goblet cell metaplasia (all *p*<0.01) (Fig 5) in the nasal mucosa and lung. Additionally, compared with cellulose group, the pectin group was found to manifest lower eosinophil infiltration and goblet cell metaplasia in the nasal mucosa (both *p*<0.01) (Figs 4B and 5B), as well as in the lung (both p<0.01) (Figs 4C and 5C).

### Dietary Fiber Reduces Levels of Serum OVA-specific IgE

Since serum IgE level reflects the relative allergic condition and enables the diagnosis of allergic disease, an increased IgE level represented severity of Th2 immune response. Therefore, we analyzed the OVA-specific IgE levels in the serum prepared from blood 24 h after the final challenge.

**Fig 6:**
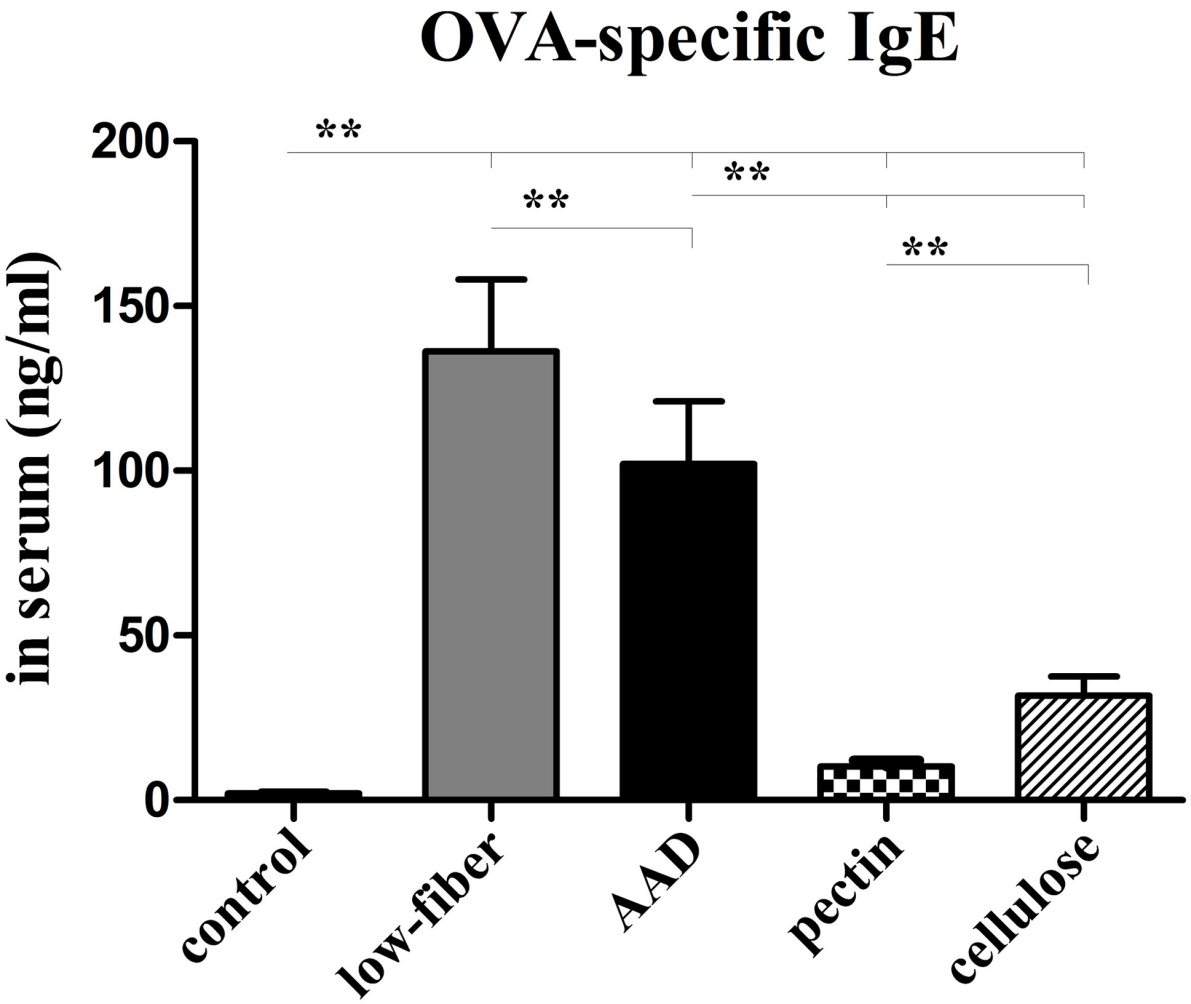
Altered serum OVA-specific IgE levels. OVA-specific IgE levels were apparently higher in low-fiber group than in the AAD model group. However, the levels in fiber-intervened mice were significantly inhibited, especially in the pectin group. Bars indicate the mean secretion ng/ml ± SEM, n = 8~10; *p<0.05, **p<0.01.

The levels of serum OVA-specific IgE were significantly higher in AAD group than in the control group (*p*<0.01) (Fig 6). Interestingly, the serum IgE levels of mice fed with low-fiber diet were further elevated, compared with the levels in AAD group (*p*<0.01). In contrast, daily dietary fiber intervention markedly suppressed the circulating IgE levels (*p*<0.01). Further, our study also revealed that fermentable-fiber (pectin) supplement significantly decreased OVA-specific IgE levels, in contrast to non-fermentable-fiber (cellulose) (*p*<0.01).

### Dietary Fiber Intervention Alters Cytokine Production

**Fig 7:**
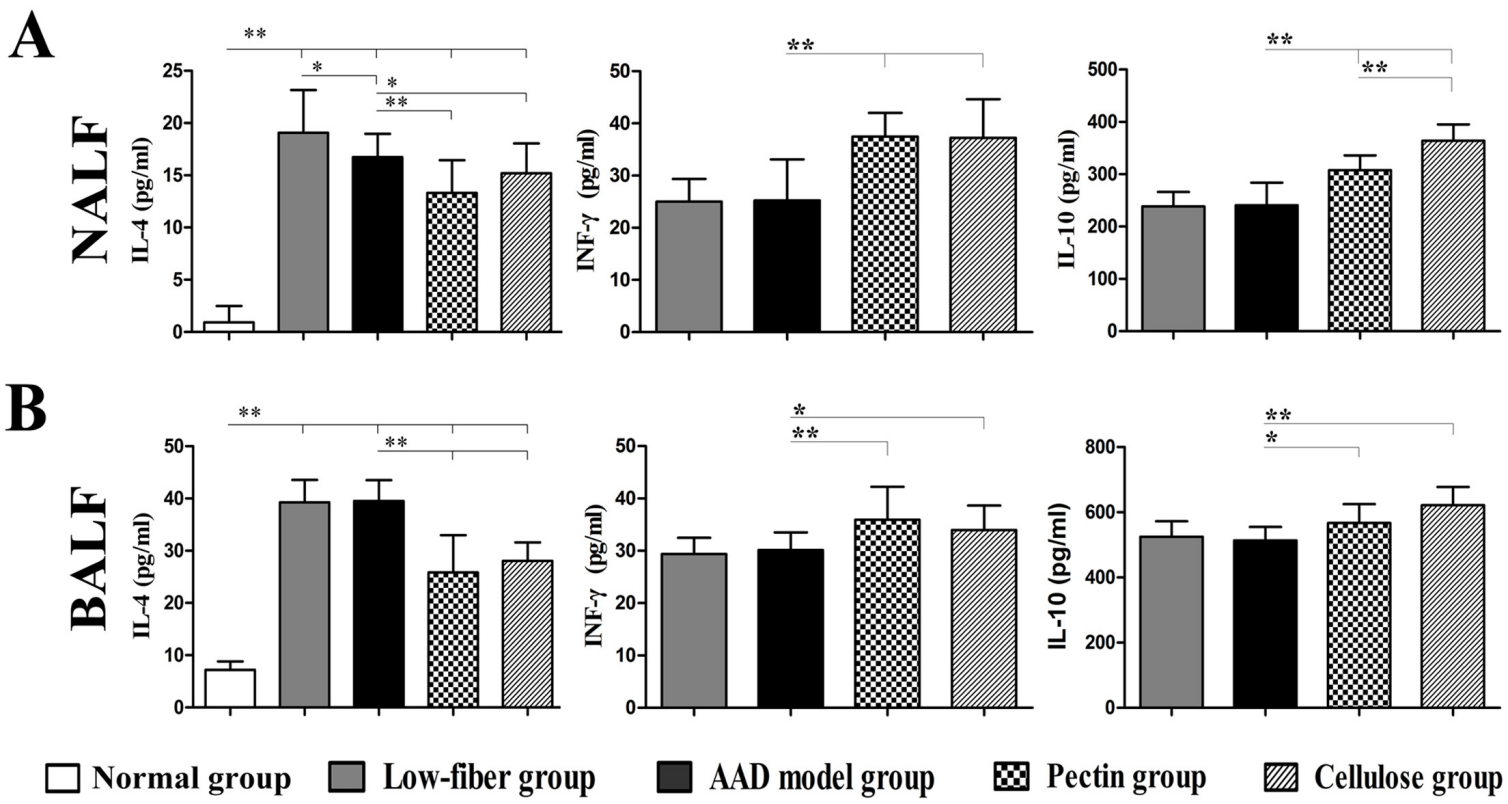
Altered levels of cytokines IL-4, IL-10 and IFN-γ in NALF (A) and BALF (B). Dietary fiber significantly inhibited IL-4 levels accompanied by increased IFN-γ levels, as well as increased IL-10 secretion. Data are represented as the mean secretion pg/ml ± SEM, n = 8~10; *p<0.05, **p<0.01 as conducted.

To further investigate the skewing of Th2-specific immune response with dietary fiber content, we measured the IL-4, IFN-γ, and IL-10 levels in both NALF and BALF (Fig 7).

NALF and BALF from AAD model group showed detectable levels of Th2 cytokine IL-4 compared with the control group (both *p*<0.01), along with a relatively higher level in NALF of the low-fiber group (*p*<0.05 for NALF). Notably, the fiber-intervened groups, especially with pectin fiber (all *p*<0.01), contained apparently low IL-4 levels compared with the AAD model group (*p*<0.05 for IL-4 in NALF from cellulose group, *p*<0.01 for IL-4 in BALF from cellulose group). However, the levels of Th1 cytokine IFN-γ in both NALF and BALF were significantly higher in fiber-intervened groups than in the AAD model group or low-fiber group (*p*<0.01 for IFN-γ in NALF, *p*<0.01 or p<0.05 for IFN-γ in BALF). Increased production of IL-10 was similarly observed in mice intervened with extra dietary fiber, compared with the AAD model group (*p*<0.05 or *p*<0.01 for IL-10 in NALF and BALF), with no significant difference in low-fiber diet group.

### Dietary Fiber Modulates Microbial Community Structure

**Fig 8:**
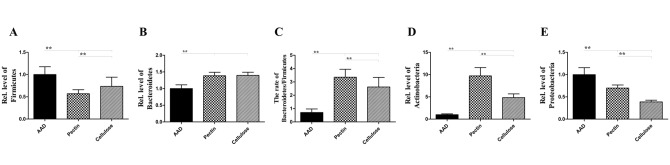
Dietary fiber modulates microbial community structure. Compared with AAD model group, the proportion of Bacteroidetes and Actinobacteria in fiber-fed group was proportionally increased, while the population of Firmicutes and Proteobacteria was distinctly reduced, compared with the decreased proportions of Firmicutes to Bacteroidetes. These changes are closely correlated with the development of allergic diseases. Data are expressed as mean relative ratio± SEM, n = 8; *p<0.05, **p<0.01 as conducted.

**Fig 9:**
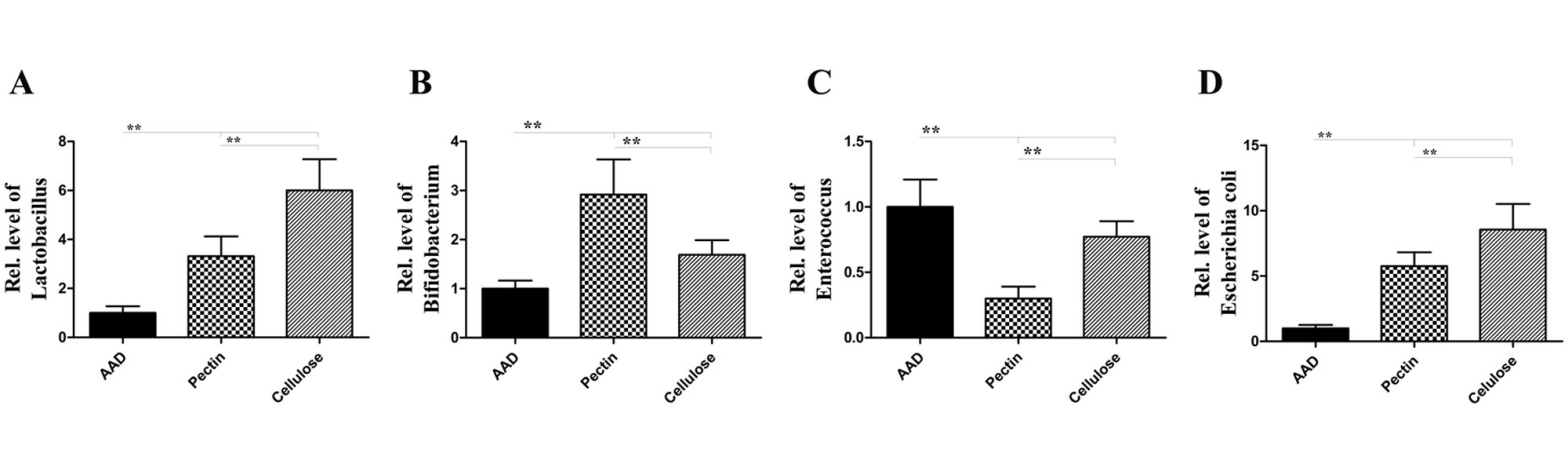
Dietary fiber facilitates the growth and proliferation of probiotic bacteria. Long-term intervention with dietary fiber significantly elevated the concentration of *Lactobacillus*, *Bifidobacterium* and *E*.*coli*, compared with the standard diet, and the level of *Enterococcus* spp was proportionally reduced. Bars indicate the mean relative ratio ± SEM, n = 8; *p<0.05, **p<0.01 as conducted.

In order to investigate the mechanisms linking dietary fiber with airway allergic disease, we extracted the total microbial DNA from fecal samples and performed quantitative analysis of bacterial populations using real-time PCR (Figs 8 and 9).

The data suggested that at the phylum level (Fig 8), the proportion of Bacteroidetes and Actinobacteria increased in fiber-fed group, compared with the AAD model group. 1.38-fold increase in Bacteroidetes and 9.71-fold increase in Actinobacteria, with pectin diet; and 1.40-fold increase in Bacteroidetes and 4.83-fold increase in Actinobacteria, with a cellulose diet (all p<0.01) (Fig 8). In contrast, the population of Firmicutes and Proteobacteria was distinctly reduced in the fiber-fed groups; 0.57-fold decrease in Firmicutes and 0.70-fold decrease in Proteobacteria fed with the pectin diet; and 0.73 -fold decrease in Firmicutes and 0.39-fold decrease in Proteobacteria with a cellulose diet compared with a standard diet (all p<0.01) (Fig 8). The increased Firmicutes-to-Bacteroidetes ratio was observed (pectin fiber, 3.82-fold increase; cellulose fiber, 2.99-fold increase, compared with mice fed with standard diet) (all p<0.01) (Fig 8).Further, at the generic level(Fig 9), data showed that, after prolonged intervention with dietary fiber, the levels of *Lactobacillus*, *Bifidobacterium* and *E*.*coli* were significantly raised: *Lactobacillus*, 3.31-fold increase; *Bifidobacterium*, 2.92-fold increase; and *E*.*coli*, 5.75-fold increase following a pectin diet, along with *Lactobacillus*, 6.00-fold increase; *Bifidobacterium*1.69-fold increase; and *E*. *coli*, 8.56-fold increase under a cellulose diet, compared with a standard diet (all *p*<0.01) (Fig 9), compared with a standard diet. However, as shown in Fig 9, the levels of *Enterococcus* spp were proportionally reduced (0.30-fold decrease with a pectin diet, along with 0.77-fold decrease with a cellulose diet, compared with a standard diet) (all *p*<0.01) (Fig 9).

## Discussion

According to ‘microflora hypothesis’, the increased epidemic of allergic rhinitis and asthma is closely associated with the shift in dietary patterns in the past decades, including reduced consumption of dietary fiber, supported by robust epidemiological evidences [7,12,14,25]. However, the regulatory mechanisms, as well as the level of impact of dietary fiber against AAD, were still unclear. Therefore, herein we developed a new mouse model with allergic rhinitis and asthma inducted by ovalbumin (OVA), based on the common pathologies and mechanisms shared in upper and lower airway [1,2,26]. The model exhibited frequent nasal rubbing and sneezing, abundant inflammatory cell infiltration into the nasal mucosa and the lung and excessive Th2 skewing of the immune responses.

Our study revealed that chronic low-fiber diet increased susceptibility to allergic airway diseases, while appropriate dietary fiber supplementation significantly suppressed the allergic inflammation of respiratory airway. As mentioned above, the reduced frequency of allergic symptoms in fiber-intervened mice was an instinctive response to remission of allergic inflammation[24]. The lower levels of eosinophil infiltration and goblet cell metaplasia in the nasal mucosa and lung, as well as the reduced numbers of eosinophil cells, further confirmed that dietary fiber strongly inhibited the induction of allergic inflammation in both allergic rhinitis and asthma.

Notably, dietary fiber exerts its protective effects observed in this work, probably, partly via regulating shift from Th1 to Th2 immune response to the OVA allergen. In order to discriminate the state of Th1/Th2 subsets in allergic airway, we assayed the levels of Th2 cytokines IL-4, Th1 cytokines IFN-γ and Interleukin-10 (IL-10) as well as OVA-specific IgE expressions by ELISA. The experimental data suggested that dietary fiber inhibited significantly the expression of Th2 cytokines IL-4, increased Th1cytokines IFN-γ levels, with the enhanced IL-10 secretion and the reduced levels of serum OVA-specific IgE. Nevertheless, the alteration of Th1/Th2 cytokines was an important indicator of the functional changes in suppressing the aberrant immune response and eosinophil inflammation in allergic diseases [27,28]. As explained by previous evidences, interleukin-10, an anti-inflammatory cytokines, restrained and even terminated the inflammation [29,30]. In this sense, the up-regulation of IL-10 expression may well be another potential mechanism of dietary fiber-mediated suppression of Th2 immune response. Furthermore, the lower level of IgE also objectively reflected the attenuation of Th2 immune response and the immune tolerance to allergic antigen [31,32]. For these results, it is reasonable to deem that this inhibitory effect of fiber supplement on eosinophil inflammation is related to the suppression of Th2 skewing of the immune response and the balance of Th1/Th2 immunity conferred by certain fiber content.

How does dietary fiber intake influence immune response? Our study implies that fiber supplements altered considerably the structure of microbial communities and the composition of intestinal bacteria, while displaying unexpected functions of immune regulation and anti-inflammatory effects. ‘Microflora hypothesis’ and relevant researches [9–13,33,34] suggested that diet habit and its effects on the gut microbiota and immune responses are increasingly likely explanations for the greater incidence of allergic and inflammatory disease in the west. According to Maslowski’s perspective[35], chronic low-fiber intake adversely affects the makeup of the gut microbiota, leading to less production of physiologically active byproducts, which are key driving factors for the increasing prevalence of inflammatory disease. Short-chain fatty acids (SCFAs), the major of fiber metabolites, have profound effects on T cells function and differentiation, which has been demonstrated by mounting body of evidence[18,19]. Trompette’s research[18]found that dietary fermentable fiber and SCFAs shape the immunological environment and influence the severity of allergic inflammation in asthmatic mouse, and the effects of propionate on allergic inflammation were dependent on G protein—coupled receptor GPR41. More recently, Thorburn’s study [19] showed that high-fiber diet or acetate-feeding induced suppression of allergic asthma by shaping microbiota and enhancing Tregs development and function, as well as altered the offspring ability to develop AAD.

Although with embedded study, the immune-modulatory effect of microbial metabolites is gradually known or accepted, owing to the multiplicity and complexity of diet-microbiota-immune system, the current understandings are still insufficient to explain completely the underlying mechanisms, remaining unknown about the interaction. But another equally intriguing finding emerged from the study is that insoluble-fiber cellulose, poorly fermented by gut bacteria, also exhibits a strong prevention and inhibiting effect of allergic airway inflammation. Apparently, it is likely to need other pathways to further explain these potential influences of fiber-diet. By the comparative analysis of the microbial colony structure among the groups, our study noted that similar to the role of readily fermentable pectin, poorly fermentable cellulose significantly improved the structure of intestinal flora by increasing relative ratio of Bacteroidetes to Firmicutes, as well as increased the count of common probiotic bacteria such as *Lactobacillus* and *Bifidobacteriumare*. Interestingly, Bacteroidetes bacteria are major producers of SCFAs and the increased ratio of Bacteroidetes to Firmicutes has also been observed in mice exposed to Western high-fat diets [9,13,36,37,38]. In addition, *Lactobacillus* and *Bifidobacteriumare*, as typical probiotic bacteria, enable the homeostasis of immune cells and decrease the susceptibility to allergic inflammation, well-supported by a mounting body of evidences [10,11,39–44]. Furthermore, the beneficial effect of E. coli against allergic diseases has also been confirmed in other animal studies [26,45–46]. As Maslowski [35] argued, if diet affects the composition of microbiota, and the microbiota regulates immune and inflammatory responses, then diet changes should have easily quantifiable effects on the immune response. For these reasons, the protection of cellulose against AAD maybe more dependent on the direct interactions of microbiota-immune system influenced by fiber-induced variations in Gut microbacteria. The inconsistent protection mechanisms need to be investigated further.

Briefly, all data indicated that both pectin supplement and cellulose supplement modulated the structure of microbial communities and altered the composition of gut microbiota effectively. These complex interactions played an essential role in the suppression of AAD.

In conclusion, based on the theories of ‘hygiene hypothesis’ and ‘microflora hypothesis’, our study, for the first time, demonstrated that dietary fiber strong inhibited the OVA-induced allergic inflammation in allergic rhinitis complicated with asthma, along with a significant modulation of the composition of gut microbiota. Our study even demonstrated that appropriate fiber intake regulated intestinal bacteria and maintained immune homeostasis, for prevention and treatment of AAD.

## Supporting Information

S1 File Table A, Absolute numbers of inflammatory cells in 1.2 ml of NALF and BALF at 24 h after final challenge. Table B, Absolute numbers of inflammatory cells in 1.2 ml of NALF and BALF at 24 h after final challenge.Table C, Eosinophil inflammation assessed with hematoxylin and eosin (HE)-stained tissue sections of the nasal mucosa and lung. Table D, Goblet cell metaplasia in alcian blue-periodic acid Stiff (AB-PAS)-stained tissue sections of the nasal mucosa and lung. Table E, Altered serum OVA-specific IgE levels Table F, Altered levels of cytokines IL-4, IL-10 and IFN-γ in NALF and BALF. Table G, Dietary fiber modulates microbial community structure. Table H, Dietary fiber facilitates the growth and proliferation of probiotic bacteria.
(XLSX)
